# Computer Tomographic Illustration of the Development of the Pulmonary Function in Bovine Neonates until the Twenty-First Day Postnatum

**DOI:** 10.1155/2013/157960

**Published:** 2013-09-19

**Authors:** Bernd Linke, H. Bostedt, A. Richter

**Affiliations:** ^1^Clinic for Obstetrics, Gynaecology and Andrology of Large and Small Animals, Justus-Liebig University Giessen, Frankfurter Street 106, 35392 Giessen, Germany; ^2^Large Animal Clinic for Theriogenology and Ambulatory Services, Leipzig University, An den Tierkliniken 29, 04103 Leipzig, Germany

## Abstract

The aim of this study was to analyze the development of the lung in newborn calves. The sample consisted of 28 Holstein Friesians calves which were examined clinically, and their chest segment was measured with computed tomography. The tests were performed on the first, sixth, and twelfth hours of life and after the first, second, and third weeks. Also, blood gases and blood counts were determined. Besides Kolmogorov-Smirnov tests, analyses of variance, *t*-tests (on a significance level of *P* < 0.05), and correlation analyses were used. The most significant changes occurred between birth and the first hour. However, there were significant differences in the gas filling between cranial and caudal and between dorsal and ventral parenchyma segments. This difference remained over the entire study period. At the end of the first week between 85 and 93% were involved in gas exchange. Only after the completion of the second week of life, the air supply was achieved throughout the whole lung. The pO_2_, pCO_2_, and pH values confirmed this. This study shows that a healthy bovine neonate needs about 2 weeks before all lung units are integrated into the gas exchange. This explains why calves in unfavorable environments often suffer from pulmonary affections.

## 1. Introduction

In many publications, mainly from the last 70 years, researchers have addressed questions regarding the developmental anatomy and physiology of the fetal and postnatal lungs. Essential research to understand the development of pulmonary tissues directly post natum was driven by the team around Barcroft [1–3]. The fundamental cross-species results of their and the following research, mainly studied in sheep and lambs, are still valid today [4–17].

Based on the present knowledge, it has generally been agreed so far that with the first breaths lung areas progressively start filling with gas, which is completed after a few hours [18–23]. Thus, the oxygenation of neonatal bodies is secured. The question, whether a full ventilationof the lungs is reached within 12 hours p. n. for healthy, vital bovine neonates, has not yet been finally clarified [24–26]. Various research teams presume due to histological findings that the lung maturation in the alveoli stage still takes place in the first post uterine phase in neonatal ruminants [16, 27–30]. Serial radiographic-sonographic studies in vivo on native vital, healthy bovine individuals could not reveal whether 12 hours after birth the pulmonary tissue is fully ventilated [31].

Therefore, the relevant question is, at which time all alveolar regions in bovine neonates fully participate in the gas exchange process. The objective was to thoroughly examine the lung regions development in bovine neonates. Using computer tomographic technology it was possible to obtain exact data expressed in Hounsfield units to describe the lung capacity of specific pulmonary layers.

## 2. Material and Methods

### 2.1. Proband Groups

For the study, 28 Holstein Friesian SB calves (HF_SB_, ♀  *n* = 14; ♂  *n* = 14) were examined. The corresponding mother cows (milk  yield > 8000 kg/year) were clinically healthy during the entire late period of pregnancy. The births took place in large boxes with straw bedding, under constant general supervision. All mother cows had shown a physiological duration of gravidity (278 ± 8 days). The calves born alive and vital (*n* = 25) derived from complication-free births. In these calves, the respiratory activity started without artificial intervention immediately after passage of the fetal thorax and abdomen out of the rima vulvae.

The status assessment via clinical surveys (ethological findings) and the modified APGAR scores for the complication-free born calves showed no deviations from the standard and an APGAR value of ≥7.5 points [32, 33]. Also, the data of the blood gas measurement ascertained before the first computer tomography examination (1 hour p. n.) showed that all vital calves were sufficiently but differently oxygenated and that the acid-base balance was stable. Moreover, there were no differences in the haemogram. In three other cases, an obstetric intervention was necessary. Those fetuses already died intrapartum (*n* = 2), respectively, one calf shortly after the expulsion (<1 minute p. n.).

The probands were divided into four groups (Table 1). The main group is group A, to which 3 control groups were allocated under different aspects.

Group A (*n* = 15) solely consisted of *mature*, eutrophic, vital calves that were declared healthy neonates based on a clinical examination directly post natum (p. n.). They underwent serial computer tomographic examinations with slight sedation until the twenty first day of life.

Group B (*n* = 3) assembled calves that died after a physiological duration of gravidity intra partum (*n* = 2) or directly after birth (<1 minute) p. n. (*n* = 1). Their data serve as an illustration of the conditions of a nonventilated respiratory tract and thus for the comparison to the computer tomography results at 1 hour p. n.

In group C there were four vital born calves, which, however, carried a life-limiting abnormality (Colonatresie, progressive opisthotonos, increasing blindness, and umbilical malformation). For animal welfare reasons, they therefore had to be clinically euthanized at different time points (first, third, fifth, fourteenth days of age) determined by their clinical status. However, as they had demonstrated normal lung function at birth, they enabled the collection of time associated histological findings of pulmonary tissue.

In vital, healthy calves built group D (*n* = 6), in contrast to group A, only a single sedation in the twelfth hour (*n* = 2) and on seventh day of life (*n* = 4) was used. This should ascertain whether the triple sedation in the first twelfth hours p. n. in calves of group A may affect or delay the ventilation of the lungs or whether this criterion could be disregarded.

The experimental design was officially verified and approved by animal welfare considerations (26/01/2009).

### 2.2. Technical Equipment

For the investigation a computer tomography trade CT PQ 2000 from Picker International (Cleveland/Ohio, USA) was used. It offers the possibility of false-color display for illustrating less and well-ventilated lung areas in the cross-sections and to use this feature in the qualitative evaluation.

The accompanying blood gas measurements were performed with the State Profiles PHOX blood gas analyzer (Waltham, USA). Furthermore, an analysis system (Haematology measuring unit Scil Vet abc, Horiba ABX Diagnostics, Viernheim, D) controlled by microprocessors served for the determination of the red and white blood count.

For the investigation were a series of CT PQ 2000 from Picker International (Cleveland/Ohio, USA) was available. There was the possibility of false-color representation of less well-ventilated ventilated lung areas in the cutting planes to visually recognize and to use this feature in the qualitative evaluation.

The accompanying blood gas measurements were performed at the State Profiles PHOX blood gas analyzer (Waltham, USA). Furthermore, the red and white blood count was determined by a microprocessors controlled analysis system (Hämatologieme*β*einheit Scil Vet abc, Horiba ABX Diagnostics, Viernheim, D).

For the animals that were available after euthanasia (group C), test material was removed evenly from four pulmonary segments to be analyzed histologically.

The samples were preserved in formaldehyde solution and enclosed in paraffin blocks. The coloration of the specimens was done using haematoxylin-eosin (H&E staining) or according to the Elastic-van Gieson method.

### 2.3. Implementation of Research

Immediately after the birth of the individual probands, a veterinary-clinical evaluation was conducted regarding ethological criteria and APGAR values. Only calves were included in the study series, which were born without complications and directly p. n. had adapted to their environment and had shown stable stamina within 30 minutes p. n. Their APGAR values p. n. ranged from 7 to 8 points. The selected probands were then laid on a special trolley without physical impairment to the CT unit. Immediately after arrival (40 minutes p. n.), the first blood was collected. Arterial blood was obtained from the Ramus intermedius medialis of the Arteria auricularis caudalis using disposable needles of the smallest dimension (Sterican 0.8 mm, Braun Melsungen, D), [34]. The blood gas analysis took place 30 seconds after the sampling. Simultaneously, a haemogram (venous blood from the vena jugularis externa) was created. Then the animal was weighed to accurately calculate the volume of the sedative. After that, the proband was given xylazine hydrochloride (0.09 mg/kg bw, xylazine 2%, Serumwerk Bernburg, D), to have a slightly sedated and relaxed calve during the measuring process without impairing the respiratory function. The proband was then stress-freely fixed on the couch of the CT scanner in sternal position with head and forelimbs stretched in cephalad, taking care that its chest is not restricted and breathing not impeded. It was achieved to secure 20 minutes without moving, hence, enabling an undisturbed measurement. The time line of the overall study is shown in Table 2.

### 2.4. Accommodation of Calves during the Study Period

Calves of group A were kept in individual pens with straw bedding and room temperature after the first CT measurement. They were given heterologous colostrum (1.5–2.0 kg) administered via an Oesophagus tube 2 hours after the initial examination. After the second and third CT passage they were again fed colostrum ad libitum from a drinking device, so that the total of colostrum within the first 12 hours of life amounted to 3-4 kg.

Throughout the measurement period, all probands were constantly supervised by a veterinarian. None of them suffered from typical neonatal diseases such as pneumonia, diarrhoea, omphalitis, or other diseases. The general well-being was never disordered and the weight gain was continuous and normal, thus, ensuring that each proband and the whole group were developing well during the first postnatal phase. 

### 2.5. Determination of Cross-Sections

To obtain consistent data of the thorax region, a proband of group B (stillborn) was fixed immediately p. n. before the main measurement series on the CT couch and analyzed section by section with the computer tomograph. The obtained results of the whole-body tomography were saved. Thereafter, the cryopreservation of this control calf was carried out in the in vivo position necessary for computer tomography. After total freezing (−20°C), a second CT scan was carried out in the same position and the data were also secured [35]. Thereby enough information for a comparison of the two series of measurements is available. The aim of this second CT examination was to define representative cross sections for the evaluation. This was color-coded in the specimen via laser beams of the CT. Then, the frozen calf was cut into sections at the marked positions. Those cross sections were documented photographically. These measurement points were linked exactly to the corresponding CT sections.

### 2.6. Data Collection and Statistical Analysis

For the measurements of the lung, two dorsal (A and C) and two ventral (B and D) quadrants were defined. The first cranial measurement position was defined as the point at which definitively lung tissue of the cranial lobes pulmonis is scanned. It was continued in a caudal direction. Due to the different lengths of the bovine neonates, the measurements in the quadrants were expanded differently.

In the first 1–3 cm of cranial measurement positions, lung parenchyma parts were partially not represented clearly in the primary studies. The range from 8 to 11 cm covered the heart area, so that in the ventral quadrants partially no measurements of lung parenchyma tissue occurred. The measured values of the last 10 cm on the *x*-axis were related to the lung-diaphragm edge. Also in this case the lung parenchyma was only incompletely represented.

In order to achieve uniformity of the displayed pulmonary tissue, data points 4 to 7 cm for the cranial area and 12–26 cm for the caudal region were used for the statistical analysis. After reprocessing the data in Hounsfield units (HU) and transferring them to Excel spread sheets they were statistically analyzed. The measurements were stabilized, weighting each value with its two neighbouring values (if existing): *X* = (*Xn* − 1 + 2∗*Xn* + *Xn* + 1). Each measured value represented a unique index value measured within the measurement, in which it was taken. In total 30.000 data were integrated in the analysis. The statistical evaluation of data was performed using the statistical program SPSS 14.0 for Windows (SPSS Software GmbH, Munich). After testing for normal distribution using the Kolmogorov-Smirnov test, descriptive statistics were carried out calculating the arithmetic mean, standard deviation, the minimum and the maximum of the Hounsfield Units of calf lung sections, the body mass, and selected blood gas parameters for the individual examination times. ANOVA for repeated measures and pair wise comparisons of means using *t*-tests and a Bonferroni correction were tested for a significance level of *P* ≤ 0.05. The correlation calculations were done using the Pearson correlation coefficient (*r*) and the coefficient of determination (*r* ^2^). The graphs were illustrated using the MS Excel 2000. 

## 3. Results

### 3.1. Data of Stillborn Calves

Using the stillborn calves that were brought to the computer tomography analysis directly after birth of group B (*n* = 3), X-ray absorption measurements of the trachea, the main bronchi, and lung parenchyma were carried out. The measurement results of the trachea with an average of 920 (816–947) HU indicate a very good gas filling in all three calves. Also, the two main bronchi were well filled with gas and the left bronchus showed −656 ± 98 HU, thus higher X-ray absorption values with the right main stem bronchus (−765 ± 86 HU) being the lowest ones.

However, the relatively good filling of the main bronchi with gas did not continue in the lung parenchyma. A marked density discontinuity in the X-ray absorption could be found between the values of the bronchial lumina and the lung parenchyma. In the cranial lung segments the HU values ranged from +40 till −133, in the caudal from +40 till −55, with mainly positive values. The calf, that showed a heartbeat immediately p. n. and was resuscitated mechanically (chest compression), exhibited air filling only in dorsocranial lung segments. It died about 1 minute p. n.

### 3.2. Development of the Degree of Ventilation in Pulmonary Tissues of Vitally Born Healthy Calves up to the Twelfth Hour of Life (Group A)

Based on the data of the stillborn calves and those of the vital probands at the end of the first hour of life, in this section, the highest reduction of X-ray absorption takes place (from ±0 to −200 to −700 HU). In the first 60 minutes of life, however, very different development of the gas exchange surface was observed.

**Figure 1:**
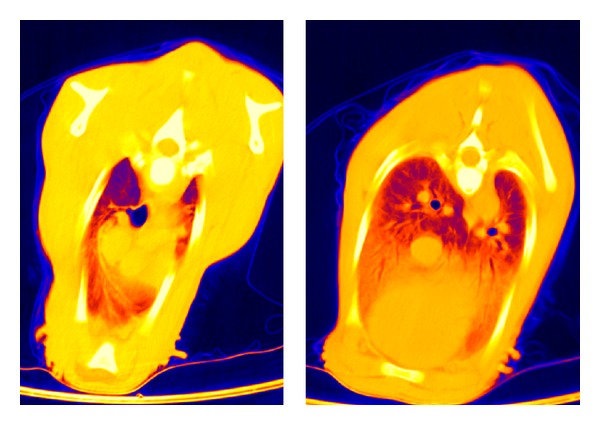
CT: thorax layer in false-color representation of a subject of group A at 1 hour p. n.

Considering a more differentiated view of the analyzed lung segments, it could be found that the dorsocranial area one hour p. n. was the best ventilated one (−570 ± 82 HU). In contrary, the gas exchange area was 14% lower in the dorso caudal section (−488 ± 58). Thus, in this segment there still was a greater lung density than in the dorsocranial pulmonary tissues (*P* ≤ 0.01). Furthermore, the calculations showed that the left dorsal lung hemisphere represented a more prominent gas filling than the right one (*P* ≤ 0.02). In the ventral lung sections, cranial right and left areas demonstrated 10% better ventilation than the caudal ones (Table 3, Figure 1). In summary it can be stated that at the end of the first hour of life, the dorsocranial gas exchange surface is characterized by higher levels of functionality than the ventrocranial (*P* ≤ 0.001), a relationship that equally applies to the dorso caudal and ventrocaudal sections (*P* ≤ 0.001).

It was striking that substantial individual differences existed with regard to the pulmonary gas exchange capacity reached in the first hour of life. This is so remarkable, because all calves were at birth and one hour later classified as vital (APGAR score ≤ 7.5) and the respiratory rate and depth of breathing 60 minutes p. n. corresponded to the standard. The individuality of lung function development is indicated by the blood gas status and is directly related to the achieved gas exchange surface. Their pH value (1 hour p. n.) ranged from 7.221 to 7.354 (7.280 ± 0.06), their oxygen partial pressure pO_2_ in kPa from 4.7 to 9.3 (7.2 ± 1.6 kPa), and their oxygen saturation (sO_2_%) from 73.2 to 94.7 (84.3 ± 7.50%).

**Figure 2:**
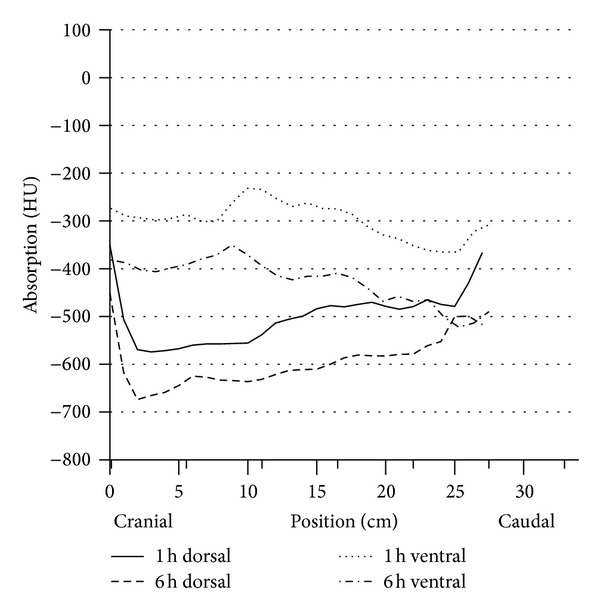
Development level of the respiration of dorsal and ventral lung parenchyma in HE of vital calves (group A *n* = 15) during the first two measurement points at the first day of life, difference *P* < 0.001, numbers in $\overset{-}{x}$.

Up to the sixth hour of life (second series of measurements), there was a further progressive development trend in the increase in the gas exchange surface area (*P* ≤ 0.001). It increased in the dorsocranial area by 15.6% and by 22.9% in the dorso caudal one (from −570 ± 82 to −659 ± 47; from −488 ± 58 to −600 ± 67 HU) (Table 4, Figure 2).

In the ventral pulmonary segments, a significant increase was also recorded (*P* ≤ 0.001). The growth rates were 37.2% and, respectively, 47.5% (from −304 ± 149 to 417 ± 133 HU ventrocranial, from −276 ± 111 to −407 ± 102 ventrocaudal). In the sixth hour of life, the same situation appeared as in the first hour: the dorsal lung hemispheres were more intensively ventilated than the ventral ones.

The analysis was extended by the question, whether some permanent influences existed regarding the individually different increase in gas exchange surface between the first and sixth hour of life. However, there was no significant relation between the gas filling ratio in the first hour of life to the animals which showed optimal or reduced values in the sixth hour. Therefore, coming from the first assessment of an individual, within the 15 five-hour span between first and second computer tomographic analyses, an individual change regarding the ventilation occurred. While those calves, which initially showed optimal values, later experienced a prolonged development, the probands with suboptimal initial values regarding the ventilation rate caught up considerably.

Neither for the first nor the sixth hour of life a correlation between the degree of increase in the gas exchange area to birth weight or to gestation duration could be calculated. Just a trend was observed considering these two parameters with regard to the HU development: a long gestation period and higher birth weight were associated in 2 of 3 cases with an optimal increase in the gas exchange surface area.

**Figure 3:**
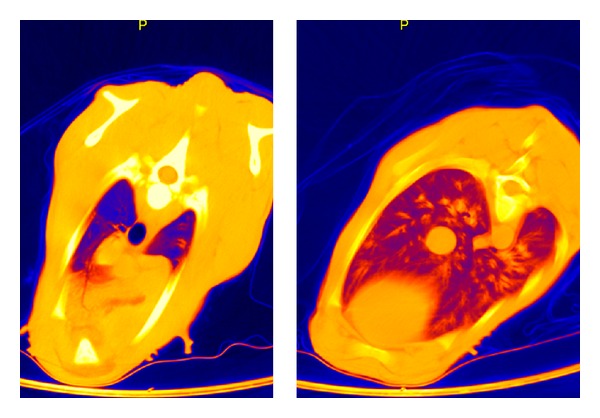
CT: thorax layer in false-color representation of a subject of group A at 12 hours p. n.

Between the sixth and twelfth hour of life (third series of measurements) no additional functional progression occurred in the dorsal lung section, but rather a protraction in terms of expansion of the gas exchange surface. The decrease in HU declined only slightly (from −659 ± 47 to −673 ± 50 HU dorsocranial, from −600 ± 67 declining to −592 ± 68 HU dorso caudal). However, the difference between the dorsocranial pulmonal tissue and the dorso caudal tissue (*P* ≤ 0.001) remained. In contrast, a gas exchange surface increase was more evident ventrally, namely, and by 14.7%, respectively, 13.0%, (from −417 ± 133 to −479 ± 135 HU ventrocranial, from −407 ± 102 to −460 ± 87 HU ventrocaudal). Despite this balancing between the dorsal and ventral lung hemispheres, considerable discrepancies existed between the maximum and minimum values (Table 5, Figure 3).

It is remarkable that between the sixth and twelfth hour of life, a protraction in the alveoli expansion processes occurs in the dorsal section. The Hounsfield units decline only slightly. The significant gradient in ventilation between the dorsocranial and the dorso caudal sections remains observable (*P* ≤ 0.001). In contrary throughout the ventral lung area, the ventilated area increases progressively up to the twelfth hour of life.

**Figure 4:**
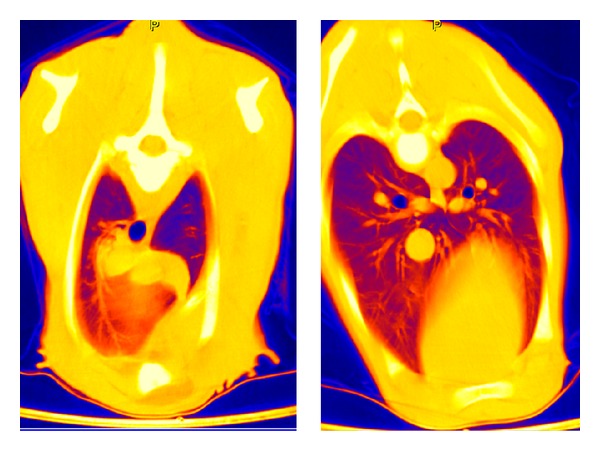
CT: thorax layer in false-color representation of a subject of group A at 1 week p. n.

**Figure 5:**
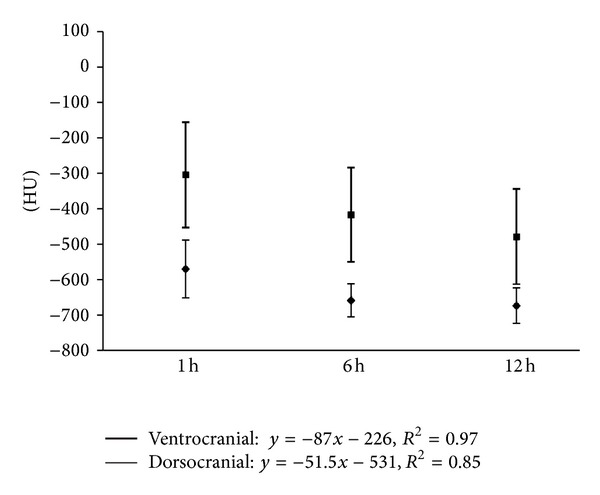
Development of the craniodorsal and cranioventral X-ray absorption values in HE of the subjects in group A (*n* = 15) at the predefined measurement times of the first day of life (⋄ = dorsocranial, □ = ventrocranial).

**Figure 6:**
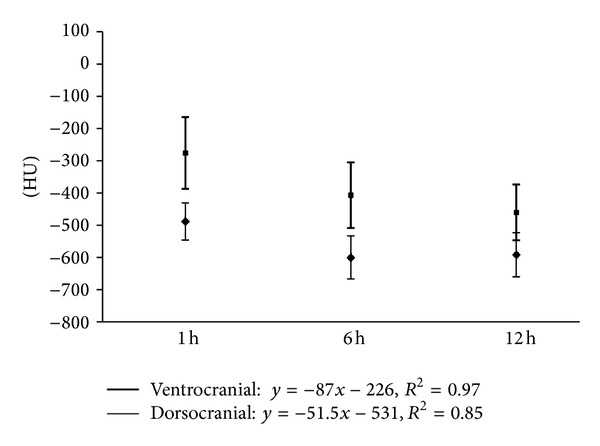
Development of the caudodorsal and caudoventral X-ray absorption values in HE of the subjects in group A (*n* = 15) at the predefined measurement times of the first day of life (⋄ = dorsocranial, □ = ventrocranial).

Nevertheless, also in the twelfth hour of life, the computer tomography in false color reproduction still showed a significant proportion of low or non-ventilated areas constantly for all probands (Figures 4, 5, and 6).

This shows that even the twelfth hour of life in bovine neonates, not all pulmonary tissue is yet functionally involved in the gas exchange process. However, all probands revealed a normal, unimpaired vitality and respiratory activity. The collected blood gas analysis values also showed no significant deviations (pH: 7.33 ± 0.06, pO_2_: 7.9 ± 2.0, kPa; sO_2_: 90.0 ± 7.8%). As it was evident that even in the twelfth hour of life the optimal expansion of the gas exchange surface has not yet been achieved, the measurements were continued. To keep the strain on the calves as low as possible and to avoid artefacts due to frequent sedation, the next CT scan was scheduled for the seventh, fourteenth and twenty-first day of life.

### 3.3. Lung Development between Twelfth Hour and Twenty-First Day of Life

All calves in group A showed a normal body development and remained free of any signs of disease until their seventh day of life. Surprisingly, on the seventh day of life, no significant changes appeared in the dorsal areas in comparison to the HU levels measured in the twelfth hour of life. The increase was only 0.3–1.8%. The gradient from cranial to caudal remained existent. In contrast, the ventral lung sections demonstrated a more pronounced reduction of the X-ray absorption values, although the differences between dorsal and ventral still remained significant (*P* ≤ 0.001). The false-color pictures indicated pulmonary segments not yet involved in the gas exchange (Table 6, Figures 4 and 9).

**Figure 7:**
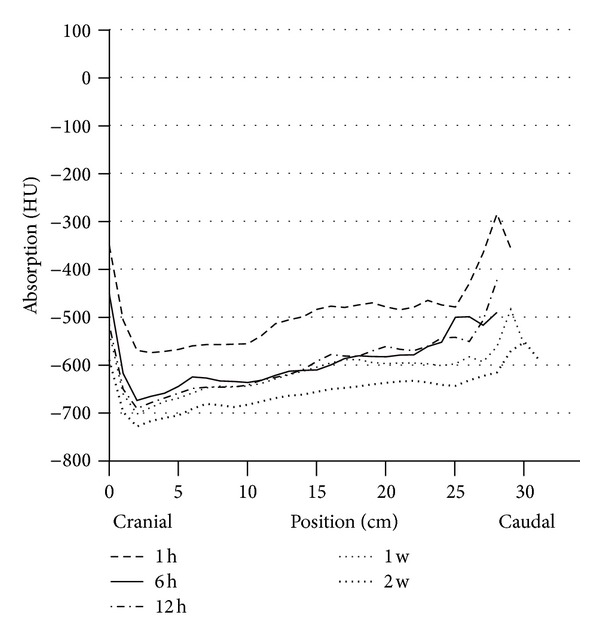
Circulation of the dorsal lung parenchyma in HE of vital calves (group A *n* = 15) during the entire research period.

**Figure 8:**
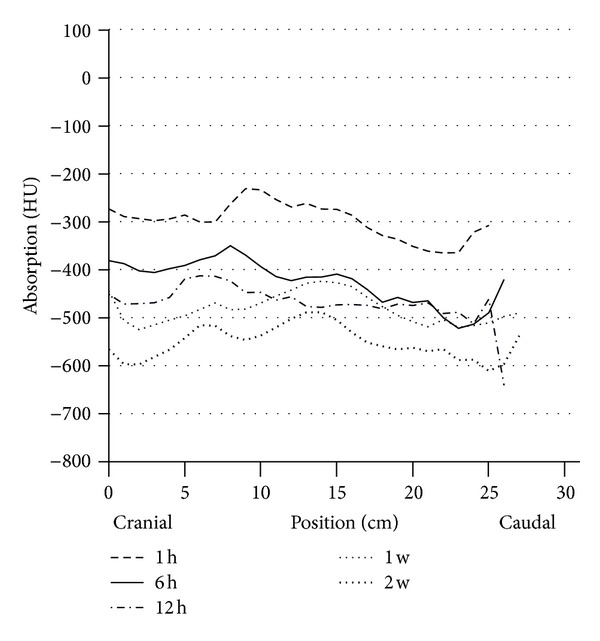
Circulation of the ventral lung parenchyma in HE of vital calves (group A *n* = 15) during the entire research period.

Only between the first and the second week of life, there was again a significant dorsal reduction in Hounsfield units (dorsocranial from −678 to −715 HU *P* ≤ 0.002, from −602 to −652 HU dorso caudal, *P* ≤ ±0.001). Even more clearly appeared the reduction of HU in the ventral region (from −501 to −581 HU ventrocranial, *P* ≤ 0.001; from −452 to −520 HU ventrocaudal, *P* ≤ 0.001) (Table 6, Figures 7 and 8).

**Figure 9:**
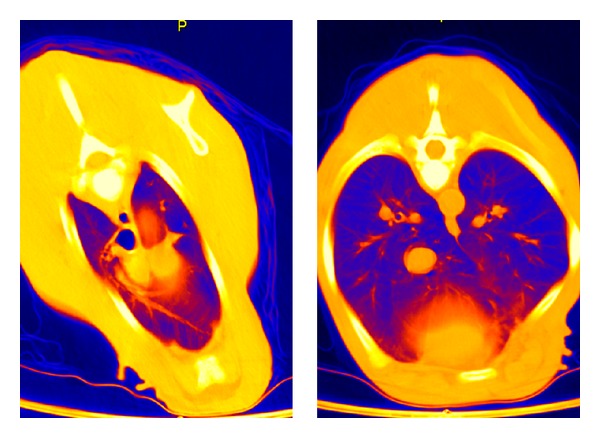
CT: thorax layer in false-color representation of a subject of group A at 2 weeks p. n.

Based on the calculated results, particularly on basis of the false-color rendering of the CT images, it is assumed that the lung tissue of bovine neonates only reached its full capacity of gas exchange around the fourteenth day of life (Figure 9). Up to this point, all developed pulmonary regions participate in the gas exchange. This cascade-like increase is reflected in the percentages of the gas exchange capacity reached at the single measurement time points (Table 7).

### 3.4. Comparative Analyses

To check whether the repeated sedation with xylazine for the computer tomography measurement on the first day of life has an influence on the lung function development, two unstressed, healthy, vital born bovine neonates were examined once at the twelfth hour of life and four on the seventh day of life (group D). Only slightly significant differences (*P* ≤ 0.03) existed compared with the data of group A in the ventrocaudal lung section at the twelfth hour of life. All other segments did not differ. On the seventh day, there was no significant difference (*P* > 0.05) in the dorsal or ventral cross sections.

### 3.5. Histological Results

Histomorphological studies were undertaken on a lung intact, vital born calf on the twelfth hour of life. The reason for euthanasia was atresia coli. The areas with gas-filled alveoli at this stage were still distributed very unevenly. In addition to well-ventilated lung areas in the craniodorsal area, sections with distinct atelectatic alveoli were mostly found in the ventral area. This is fully consistent with the CT findings. The progression of the air filling within the segments was demonstrated through the confluence of focal atelectatic and focal ecstatic alveoli sections. Therefore, at the twelfth hour of life differently ventilated areas existed next to each other. Completely ectatic areas were located next to atelectatic ones, particularly frequently in the ventral segment.

Lung tissue of two neonates at the age of 6 and 8 days p. n. was also available for histomorphological studies. Although they were born vital, they showed abnormalities not associated with the respiratory tract, which resulted in euthanasia. In the dorsal lung segments, regular alveoli septa architecture was found. In the ventral sections, however, there were still atelectatic areas that existed side by side to ectated ones. This is also reflected in HU values. However, they were strictly separated by lung septa. This suggests that the ventilation of the alveoli happens segmentally and progressively within this process.

Also, the lung tissue of a blind-born calf, which was slaughtered on the fourteenth day of life, was looked at histologically. The histological picture showed age-appropriately developed, delicate intralobular septa with inconspicuous vessels. In all regions, fully ventilated alveoli with regular outlines were found. There were no histologically reproducible differences in the levels of the gas filling process between the cranial and caudal and only slight ones between dorsal and ventral sectors. These findings thus complied with the CT results.

### 3.6. Blood Gas Analysis

**Figure 10:**
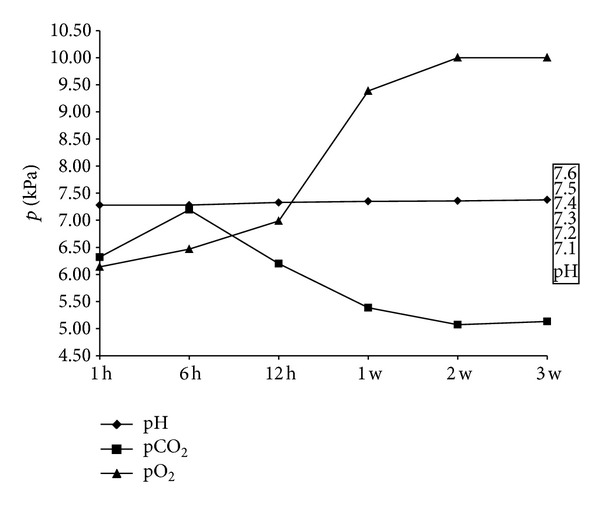
Change of arterial blood-pH; pCO_2_ and pO_2_ values of subject group A (*n* = 15) within the whole research period (values in *x* + *s*).

The pH values in arterial blood developed progressively from the first until the twelfth hour of life. Later, there was only a modest increase in blood gas analysis parameters (first hour of life pH 7.280 ± 0.06 kPa, min. 7.221, max. 7.354; twelfth hour of life 7.330 ± 0.06 kPa, min. 7.225, max. 7.419; third week 7.380 ± 0.04 kPa min. 7.354, max 7.483). The oxygen partial pressure (pO_2_) rose up protractedly until the first week, but then jumped up rapidly. Parallel relations existed in the oxygen tension (SO_2_) (Figure 10). The pCO_2_ tension on the other side declined abruptly after the sixth hour of life.

## 4. Discussion

The fetus is subject to an unstable oxygenation during parturition, on the one hand, triggered by the birth-associated uterine contractions that lead to a temporary, partial oxygen deficiency in both the myometrium and the fetus. On the other hand, already at this time the intensive fetomaternal connections loose in bovine placental tissue. 

Both conditions determine that—depending on birth length—the fetus suffers from a more or less severe reduction in oxygen supply and is subject to a protraction of the carbon dioxide output. Hypoxia and hypercapnia are the results. This subpartial situation is probably balanced with an O_2_-saving feature, which temporarily leads to a consistently high tissue oxygen supply only in the heart muscle and the brain; other fetal body parts are downrated for a short term [36–38].

This condition must, however, remain limited in order not to induce any tissue damage. With the break of the umbilicus, the cardiorespiratory adaptation to the changing life conditions must immediately start post natum, to secure the functionality of all organs and the increased need for oxygen for the postnatal onset of metabolic and somatotropic processes.

The time at which the pulmonary tissue in neonates is fully integrated into the diffusion process to adjust the O_2_ supply to these circumstances is evaluated differently. A number of authors assume that, with the first breaths, the ventilation of the lungs is deemed completed [12, 20, 21]. Other working groups assume, according to their findings, that this process is completed nearly fully with the twelfth hour of life [15, 16, 39–42]. Basis of all these definitions are studies that have used predominantly ovine and also, caprine and canine neonates. Based on these results, cross-species conclusions were drawn. A mandatory determination for bovine neonates, however, to calculate at what point p. n. an optimal gas exchange surface is reached, could not be made because of heterogeneous reasons [17, 25–27, 29, 31, 43]. However, based on the present studies, for bovine neonates, a longer period of time is needed regarding the development of diffusion capacity. It has to be considered that these results are the results of in vivo serial examinations of consistently healthy and vital collective of bovine neonates, balancing the individuality in the postnatal lung development calculations to fixed size that always exists.

Two very important essential processes in the first few seconds and minutes of life are closely linked: the cardiorespiratory transition and the induction of the first breath [1, 6–8, 12, 22, 23, 44]. Controversially discussed is whether the first air intake in the trachea-bronchial section of respiratory system happens actively or passively [20, 21, 42, 45]. In general opinion, it requires a so-called opening pressure, which must be present in vital neonates only to cope with the first air intake.

The data of the stillborn neonates in this study's measurements show that the first aspiration of air has to be interpreted as a passive process. Their trachea and the two main bronchi showed the same CT values at −900 HU as the vital calves, which indicate a pronounced air filling, regardless of the degree of vitality. The process that has to be characterized as passive can be explained by the birth situation. With the passage of the thoracic segment of the osseous and soft birth canal an oval shaping and compression of the thorax is associated. With expulsation of the head and thorax through the rima vulvae this tension drops abruptly alike in the living and stillborn fetus. The abrupt change in pressure conditions leads to relaxation and elongation of the thorax. This allows for the passive aspiration of air into the upper respiratory tract, if it is not blocked by mucus, even before the first active breath.

Furthermore, it is not accurate to state that already the first breaths of bovine neonates lead to a complete unfolding of the lung tissue in all segments. Rather, it can be derived from the data that at the end of the first hour of life the left and right lung sections are involved in gas exchange process only at 50–80%. The dorsal sections show a significantly (*P* ≤ 0.001) better ventilation (74–80%) than the ventral ones (50–52%). However, the individual differences are striking, although all calves were born without complications and had developed normal breathing. This is indicated by the Apgar scores and the blood gas analyses. 

Connected to this, no secure relations exist regarding birth weight or gestation duration in the proband collective. Also, the duration of labor corresponded to the physiological norm, so that this factor—if considerably prolonged—can lead to negative conditions of pH and acid-base values and cannot explain the individuality at the end of the first hour of life [46]. Therefore, there must be other reasons for the segmental different gas fillings of the lung tissue. Some starting points, maybe subjective ones, are offered by the clinically ethological observations. Strict observations of neonates show that the respiratory activity can be very irregular until the thirtieth minute of life [47]. In some of calves born alive, the active breathing is shallow and discontinuous, with short apneic periods. In others, shortly p. n. rhythmical breathing with accompanying deep breaths can occur. 

The latter calves also rise earlier and attain a stable standing position. Calves with shallower breathing remain longer in the sternal recumbent position [47]. These protracted lying intervals within the first 30 minutes of life are not to be described as abnormal but are still within the given range of variation in the stabilization of vigilance. According to Dean [48], the position of the neonate influences the intensity of the respiratory activity immediately post natum. Likewise, in this context, the published results of Uystepruyst and colleagues [30] indicate the importance of postnatal lying position. Resistance and reactance depend on the position of bovine neonates. This would reinforce the opinion that the form and duration of postnatal lying position in the early phase p. n. carry out an influence on the development of pulmonary gas exchange surface, at least in the short term. So, the shallow breathing due to the position could be a reason for the unbalanced gas filling, especially in the dorsal lung segments. The restrictive ventilation of ventral pulmonal regions can be rather explained by gravity and insufficient squeezing out of alveoli fluid intrapartum, caused by the intravaginal-compression processes intrapartum. To that extent, position and gravity-induced conditions are partly responsible for the development of individuality in the process of lung ventilation until the first hour p. n.

Beside these ethological and lying position-related criteria, a number of other factors need to be considered for the interpretation of a not fully ventilated lung at the end of the first hour of life. It can be assumed that the transition from cardial right-left shunt to the left-right shunt and the associated increase in blood flow intensity through the lung tissue are not completed equally in all newborn individuals until the end of the first hour of life. This seems to be connected to the gradually decreasing lung resistance. Although Cassin et al. [8] have found that in sheep fetuses the lung resistance gradually decreases until birth, which correlates with increased metabolism and growth expression of lung tissue, it can be assumed that immediately post natum the minimum value is not yet reached [49]. The work of Varga et al. [43] and Uystepruyst and coauthors [30] points out that according to their measurements with a pulse oscillation system (IOS) in bovine neonates within the first hours reveal an increased resistance value (R5HZ to R35HZ), which only decreases in the following hours. In contrary to that, the reactance value increases.

Furthermore, it should be noted that the resorption processes in the cell layers and the lymphatic drainage of the residual alveolar fluid depend on two factors. First, the blood flow capacity of the lung tissue, gradually optimizes post natum [9, 50–53]. Second, various working groups have found that fetal bovine lung tissue is subject to postnatal “localization and gestation-related” discontinuous maturation processes [25, 27, 54]. Regarding this, the formation of II pneumocytes plays an essentially important role. Basic data in bovine fetus are stated by Schoon [25]. He showed that from the two-hundredth day of gestation, the area of prospective air-guiding systems increases not only until birth but even later on. He could not state an exact limit for this procedure because his morphological investigations only took until the third day of life. De Zabala and Weinmann [27] had indicated earlier that the alveolar maturation lasted beyond the fetal period and might extend up into the early postuterine period. Evidence for this assumption could be found in the experimental studies on fetal sheep and lambs by Flecknoe et al. [16, 55]. These authors showed that in the last trimester of gestation of sheep fetuses, the sum of the alveolar endothelial cells (AEC) of type II increases disproportionately. In contrary, the amount of type I AEC stays on the same level until parturition. It is an important finding that the quantity of type II AEC increases post natum, probably until the second week of life, whereas the cell collective AEC type I decreased slightly. According to the computed tomography findings this would be a further indication why the optimum in terms of Hounsfield units (HU) is only achieved around the fourteenth day of life.

In this context, the formation of the antiatelectase factor by pneumocytes II, closely associated with the synthesis of phosphatidylcholine (Ptd Cho), is of great importance Yu et al. [56] and Pérez-Gil [57]. This factor stabilizes the alveolar wall, thus allowing the gas exchange processes, Johansson and Curstedt [58] and Ridsdale and Post [17]. According to latest research, the phosphocholine cytidylyltransferase *α* plays a key role in these processes [59]. It is not assumed to be involved in the proliferation processes or in the differentiation processes of the lung epithelia. The assumption exists that the synthesis of Ptd Cho does not occur synchronously in all alveoli, but in cascades after birth within the intact alveoli, especially in those which still do not fully possess maturity structures.

The computer tomography and the resulting calculations show contradictions. The comparative data from the test series until the third week of life show that in the twelfth hour of life only 90–95% of the dorsal, but, interestingly, only 81–89% of the ventral, lung parenchyma are filled with gas. Interestingly, between the sixth, and twelfth life hour only little further increase in ventilation capacity was reached in the dorsal lung parenchyma, whereas in the ventral region this could be elicited (*P* ≤ 0.03). The differences in overall development from the first to the twelfth hour of life between craniodorsal and cranioventral lung sections are shown by the correlation factors *r* = 0.85 and *r* = 0.97, as well as between the caudodorsal and caudoventral regions (*r* = 0.69 and *r* = 0.95).

To explore whether values determined at the twelfth hour of life depict the maximum HU situation, the data collection was extended beyond this period. In order not to provoke artificially induced effects, the calves were again examined with a CT scan at longer intervals, at the seventh, fourteenth, and twenty-first day of life. Surprisingly, it emerged at the beginning of the second series of measurements; the HU had changed only slightly between the dorsal and ventral lung parenchyma regions from the twelfth hour of life until the seventh day of life. The gas exchange capacity reached 93%, respectively, 87%. Only the gas exchange surface area in the caudo ventral area had increased. Summing up, it can be noticed that even on the seventh day of life, the maximum gas exchange capacity was not yet reached. This result must be interpreted against the background that all probands remained free of disease in the meantime, which showed a progressive development trend, and their blood gas analyses and haematological data were in the normal range. There is currently only one comparable study on morphological basis, which can be used to interpret these findings [29]. Based on their histological examinations of individual objects performed at different time points p. n. the authors found that “… alveolar surface area and total number of alveoli increased significantly with increasing age.” This is consistent with our own histological and computer tomography findings. Together, this would be one possible explanation for the effect that with increasing body size in the early postnatal period gradually the single sections of the lung take part in the ventilation process. The inoculation of the alveoli in the fetal alveoli into the gas exchange process obviously occurs segmentally in bovine neonates. This process happens in a first phase until the twelfth hour of life and in a second one between the seventh and fourteenth day of life. In the intervals between those time points, certain stagnation occurs. The configuration change of the thorax surely plays a role. It changes its shape from highly oval to a compressed circle within that time. Thus, the possibility of expansion mainly of the ventral pulmonary tissue is given [35].

The cascade-like increase in pulmonary tissue involved in gas exchange is comprehensible considering that the lung volume and thus the gas exchange capacity correlate to the body weight in neonates. The weight gain of the probands within the measurement period (0–21 days p. n.) was 16.5%. It is assumed that in the course of maturation of fetal alveoli, representing a certain reserve, a gradual participation of pulmonary regions in the overall lung function process occurs. This is also an indirect result of the experimental study by Castleman and Lay [29]. This should be considered for the interpretation of blood gas values. It should be noted that there have been few blood-gas analysis measurements of newborn calves, bearing in mind that in the relevant publications, data collection does not continue until the third week of life [30, 43, 60–62].

Based on the data presented here, it can be noted that the pO_2_ value increases slowly up to the first week of life, then shows a progression between the first and second week of life, and then remains on a high level. Similar conditions exist for the SO_2_ levels. The CO_2_ tension decreases with a negative correlation [35]. This curve trend shows that with increase in body mass the O_2_ demand increases and, as compensation, the gas exchange volume can increase in this phase of development through activation of pulmonary tissue sections that were not yet ventilated. The latter can be interpreted as a retrievable reserve that allows the progressive increase in the metabolic response by increasing the O_2_ supply in the early postnatal period. This happens in parallel to the maturation of the pneumocytes II population and to the increase in surfactant synthesis. Additionally, the intense intra-alveolar fluid absorption primarily from the gravity-dependent ventral sections and the changing configuration of the thorax immediately p. n. until the second/third week of life should be considered as accompanying components.

## Figures and Tables

**Table 1:** Proband groups (*n* = 28).

Group	*n*	Status p. n.	♀	♂
A	15	Vital	9	6
B	3	Dead	1	2
C	4	Vital	2	2
D	6	Vital	2	4

**Table 2:** Time line of the overall study for vital, healthy calves (group A: *n* = 15).

Time p. n.	1st h	6th h	12th h	7th day	24th day	21st day
CT	+	+	+	+	+	+
Blood gas analysis	+	+	+	+	+	+
haemogram	+	−	+	+	+	+

**Table 3:** X-ray absorption values of lung parenchyma in HU of 15 vital calves in the first hour p. n. (measured data of the dorsal and ventral lung segments).

Lung section	*n*	$\overset{-}{x}$	±*s*	Minimum	Maximum
A (dorsocranial)	60	−570	82	−718	−389
C (dorsocaudal)	135	−488	58	−607	−328
B (ventrocranial)	60	−304	149	−567	−328
B (ventrocaudal)	131	−276	111	−478	−56

**Table 4:** Development of X-ray absorption values (HU) in the lung parenchyma of 15 vital calves comparing first and sixth hours of life.

Lung section	Measuring time (h)	*n*	$\overset{-}{x}$	±*s*	Minimum	Maximum
A	1	60	−570	82	−718	−389
(dorsocranial)	6	60	−659	47	−753	−551
C	1	135	−488	58	−607	−328
(dorsocaudal)	6	135	−600	67	−716	−367
B	1	60	−304	149	−567	−56
(ventrocranial)	6	60	−417	133	−631	−16
D	1	131	−276	111	−478	−556
(ventrocaudal)	6	134	−407	102	−593	−83

**Table 5:** Development of X-ray absorption values (HU) of the lung parenchyma of 15 vital calves (group A) compared between the sixth and twelfth hour of life.

Section of analysis	Measurement time p. n. h	*n*	$\overset{-}{x}$	±*s*	Min.	Max.
A + C	6	60	−659	47	−753	−551
Dorsocranial	12	60	−673	50	−751	−570
A + C	6	135	−600	67	−716	−367
Dorsocaudal	12	135	−592	68	−712	−307
B + D	6	60	−417	133	−621	−16
Ventrocranial	12	60	−479	135	−656	−23
B + D	6	134	−407	102	−593	−83
Ventrocaudal	12	134	−460	87	−650	−173

**Table 6:** Percentile development of the gas exchange capacity, measured by detectable HU in the first 3 weeks p. n.

Lung section	Measurement time				
	1 h	12 h	7 d	14 d	21 d
Dorsal	77	92	93	99	100
Ventral	52	85	86	98	100

**Table 7:** X-ray absorption values (HU) in the pulmonary tissue of calves in group A comparing the seventh and fourteenth day of life.

Measurement section	Measurement time p. n. hour	*n*	$\overset{-}{x}$	±*s*	Min.	Max.
A + C	7	60	−678	54	−779	−531
Dorsocranial	14	60	−715	31	−766	−634
A + C	7	135	−602	50	−702	−461
Dorsocaudal	14	135	−652	34	−727	−572
B + D	7	60	−501	54	−644	−290
Ventrocranial	14	60	−581	64	−719	−430
B + D	7	135	−452	111	−588	−46
Ventrocaudal	14	135	−520	92	−652	−110
